# A case report of a patient with inoperable primary diffuse leptomeningeal melanomatosis treated with whole-brain radiotherapy and pembrolizumab

**DOI:** 10.1097/MD.0000000000028613

**Published:** 2022-01-21

**Authors:** Patrik Palacka, Jan Slopovsky, Marek Makovnik, Karol Kajo, Jana Obertova, Michal Mego

**Affiliations:** a 2^nd^ Department of Oncology, Faculty of Medicine, Comenius University, Bratislava, Slovakia; b National Cancer Institute, Bratislava, Slovakia; c Department of Radiology, National Cancer Institute, Bratislava, Slovakia; d Department of Pathology, St. Elisabeth Cancer Institute, Bratislava, Slovakia.

**Keywords:** pembrolizumab, primary diffuse leptomeningeal melanoma, whole-brain radiotherapy

## Abstract

**Rationale:**

Primary diffuse leptomeningeal melanomatosis (PDLM) is a rare disease that affects melanocytes in the leptomeninges. There is very limited data on the efficacy of immunotherapy in this setting.

**Patient concerns:**

A patient (23 years old) was diagnosed with PDLM. Histologically, atypical melanocytic cells were also observed.

**Diagnosis:**

Immunohistochemistry showed positivity for S100 protein, NKiC3, and vimentin, and negativity for Melan-A and HMB-45, with a proliferation index of 30%. Extracranial disease was excluded using dermatological and other examinations, including positron emission tomography/computed tomography with ^*18*^F-fluorodeoxyglucose.

**Interventions:**

The patient was treated with whole-brain radiotherapy (10 fractions to a total dose of 30 Gy) concomitantly with pembrolizumab and then continued with immunotherapy until disease progression with a maximum effect of partial remission on magnetic resonance imaging scans.

**Outcomes:**

Progression-free survival was 6.0 months and overall survival 6.5 months.

**Lessons:**

This is one of the few case reports of an adult patient with this rare malignancy being treated with a programmed death-1 inhibitor with partial response. Immunotherapy in metastatic PDLM may be a reasonable therapeutic option.

## 1 Introduction

Primary diffuse leptomeningeal melanomatosis (PDLM) is an extremely rare malignancy, accounting for approximately 1 case per 10 million individuals.^[1]^ Leptomeningeal melanomatosis describes primary melanocytic central nervous system (CNS) lesions, which have spread to the leptomeninges. This disease stems from melanocytes in the subarachnoid space that travel from the neural crest during embryonic development.^[2]^ Diagnosis is usually delayed owing to non-characteristic symptoms, and the condition is often misdiagnosed as other diseases, including meningitis and vasculitis. Due to its rarity, there are no standard treatment regimens, and most of the treatment data are derived from published case reports of malignant melanoma with metastases to the CNS. Recently, a case report published by Krpan et al^[3]^ demonstrated the efficacy of pembrolizumab in combination with radiotherapy, where stabilization of the disease was achieved for 2 years.

In this case report, we present a patient with PDLM treated with whole-brain radiotherapy (WBRT) concomitantly with pembrolizumab until disease progression. Progression-free survival was 6.0 months and overall survival 6.5 months. No treatment-related toxicities of grade ≥3 were observed.

## 2 Case report

In January 2019, a 23-year-old man was admitted to the Department of Neurology for an abrupt onset of progressive headache with subsequent development of diplopia 1 month later. Papillary edema was observed during the examination. Magnetic resonance imaging (MRI) of the head revealed 2 striped masses on the left side of the corpus callosum. The cerebrospinal fluid (CSF) showed no pathological features. After a pulse of methylprednisolone, the patient's difficulties subsided. In January 2019, the patient was readmitted for recurrence of headache and visual disturbances. MRI of the head revealed amplification of the perineural fluid around the optic nerves. Due to increased intracranial pressure, the patient underwent insertion of a lumbo-peritoneal shunt, which, due to malfunction, was later replaced by a ventriculoperitoneal shunt. In May 2019, as difficulties progressed, the patient underwent contrast-enhanced MR angiography and MRI to exclude vasculitis as the main source of symptoms. Magnetic resonance angiography showed no signs of vasculitis; however, diffuse meningeal enhancement was observed in both the supratentorial and infratentorial regions and around the optic nerves.

In July 2019, the patient was admitted to the Department of Neurology. CSF assessment showed elevated proteins (21.30 G/L) and S100 protein positivity (23.79 ųG/L). Subsequently, a biopsy of the lesions was performed. Histologically, there was neoplastic proliferation of melanocytic cells with immunohistochemically proven expression of S100 protein, vimentin, and NKiC3 protein. All cells tested negative for melan-A and HMB 45. The proliferation index was 30%, and genetic markers (BRAF V600E mutation and microsatellite instability) and programmed death-ligand 1 (PD-L1) expression were negative. Therefore, a preliminary diagnosis of malignant melanomatosis was made. Extracranial disease was excluded on positron emission tomography/computed tomography with ^*18*^F-fluorodeoxyglucose.

**Figure 1:**
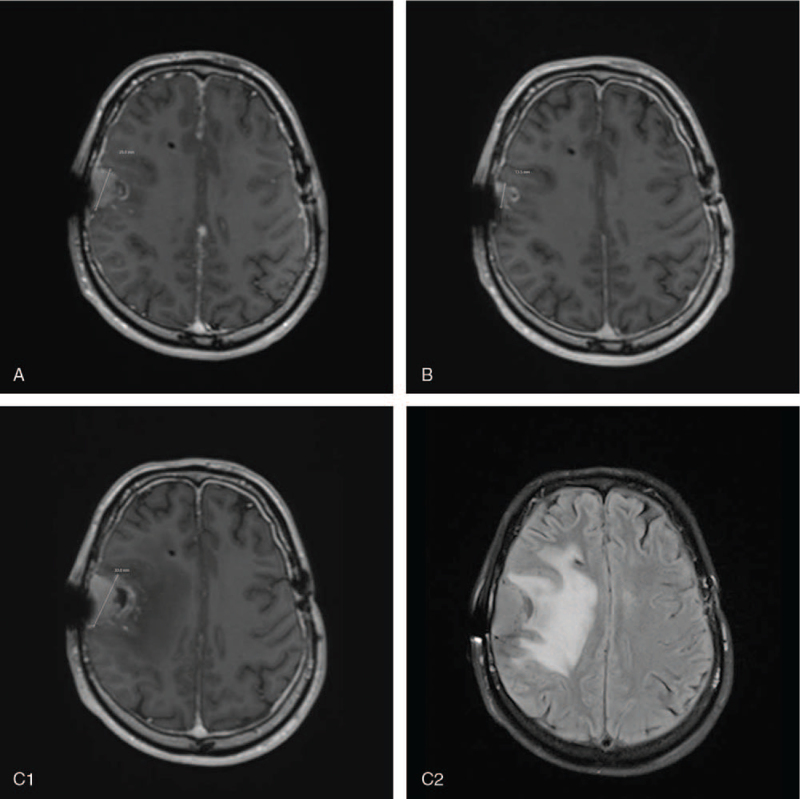
Axial post contrast T1-weighted magnetic resonance imaging showing enhanced extra-axial lesion in the region of right parietal lobe (A–C1). A and B show post chemotherapy tumor size regression. C1 shows the axial T1-w MRI tumor size progression, with a diffuse hyperintense area in the right hemisphere denoting extensive edema, as shown on axial T2 - weighted (TIRM) magnetic resonance imaging (C2).

After the initial diagnosis, the blinded patient was admitted to the 2^nd^ Department of Oncology, Bratislava, Slovakia, for the initiation of antitumor treatment. The patient was repeatedly assessed by a dermatologist who did not find melanocytic lesions on the skin and mucus membranes. No melanocytic lesions were found on gastroduodenoscopy or colonoscopy, further supporting the diagnosis of primary melanocytic malignancy of the CNS. In August 2019, WBRT of the CNS was initiated up to a total dose of 30 Gy. After the first dose of radiotherapy, 200 mg of pembrolizumab was administered every 3 weeks. Overall, the patient received 4 doses of pembrolizumab with partial remission of CT (Fig. 1B). In February 2020, disease progression was observed (Fig. 1C). Owing to poor performance status, this patient became a candidate for best supportive care after disease progression on immunotherapy and subsequently died on March 1, 2020. Fatigue, the only grade 2 adverse event associated with pembrolizumab monotherapy, was observed.

## 3 Discussion

Primary malignant leptomeningeal melanomatosis is an extremely aggressive form of nonmetastatic invasion of the leptomeninges by malignant melanocytes. Its rarity and nonspecific symptoms account for its late diagnosis. The initial presentation of malignant meningeal melanomatosis is non-specific and stems from its effect on various parts of the brain, either from locoregional infiltration or involvement of cranial nerves. A systematic review by Baumgartner et al^[4]^ of 26 case-studies identified increased intracranial pressure as the source of most of the symptoms, including headache, nausea, and vomiting, in >56% of subjects. Lepteningeal enhancement is usually observed on CT scans. PDLM on MRI scans is usually hyperintense on T1-weighted images and iso- or hypointense on T2 weighted images. CSF analysis can reveal elevated proteins, atypical cells, and reduced glucose concentrations. However, the findings are often negative, and repeat lumbar puncture is necessary.^[4,5]^

The mainstay of diagnosis is biopsy followed by histological examination. Immunohistochemically, the tumor cells were positive for S100 protein, melan-A, and HMB-45, and negative for cytokeratins.^[6]^ However, in our case study, the cancer cells were not immunoreactive against HMB-45 and Melan-A. HMB45 negativity does not rule out the diagnosis of PDLM,^[7]^ as its immunoreactivity has been demonstrated in about 86–97% of cases.

Surgery is the best treatment option for nodular lesions.^[6]^ Mostly, the treatments for this disease comprise recommendations stemming from the treatment guidelines for malignant melanoma. BRAF inhibitors can be used for BRAF-mutated tumors, although the incidence of BRAF mutations in PDLM is low.^[7]^ Conventional cytostatic treatment with TMZ or vincristine did not show significant efficacy. The response rate to dacarbazine in patients who underwent surgical resection are 16–20%.^[8]^ Other chemotherapeutic agents and immunotherapies such as interferon alpha-2b did not show significant efficacy.^[9]^ Median overall survival of patients with PDLM was reported to be 4 months (range 0–12 months) from diagnosis.^[4]^

In subjects with metastatic malignant melanoma of the CNS, immunotherapy based on checkpoint inhibition (anti-programmed death-1 or anti-CTLA-4) has shown marked activity in recent years. Single-agent immunotherapy is effective, although higher intracranial response rates were observed with combination treatment.^[10,11]^

There are very few data on the efficacy and safety of immunotherapy for PDLM. Recently, a case study published by Krpan et al^[3]^ showed the efficacy of concomitant whole-brain radiotherapy with pembrolizumab in a patient after partial resection of the tumor, with the effect of disease stabilization for 2 years; however, the price of this was progressively worsening mental status. Another case-study presented in the aforementioned review published by Baumgartner et al^[4]^ described a pediatric patient with PDLM who was treated with combination immunotherapy with nivolumab and ipilimumab. Ipilimumab was initiated after 2 doses of nivolumab because treatment with nivolumab showed only a mixed response. Combination therapy led to marked clinical improvement with normalization of CSF findings. However, it is worth noting that this particular patient was pretreated with alternating therapy of intrathecal topotecan and cytarabine, as well as with trametinib from the time of diagnosis. Another case study by Fujimori et al^[12]^ presented a 37-year-old patient in whom a diagnosis of PDLM harboring a *BRAF* mutation was initially treated with vemurafenib and WBRT without marked efficacy; after 23 days, treatment was switched to second-line nivolumab, with disease stabilization within 2 months. The overall survival in this particular case was 5.5 months. Some malignancies such as breast cancer metastasizing to the CNS have to be treated with intrathecal chemotherapy or hormonal therapy,^[13]^ however, immunotherapy has the advantage of good penetration into the CNS; therefore, it is a valuable option for the treatment of CNS metastatic diseases.^[14]^

This case report describes a patient with *BRAF* non-mutated and PD-L1 negative PDML with pembrolizumab and concomitant WBRT resulting in partial remission of malignancy on MRI scans with a duration of response of 6 months.

In summary, this case report adds to the scarcity of data regarding the efficacy of checkpoint inhibitors in the treatment of primary malignant meningeal melanomatosis. Therefore, immunotherapy might be of value to patients with this rare and aggressive disease. To the best of our knowledge, this is the first case report of a young patient with BRAF non-mutated, PD-L1 negative inoperable PDLM treated with pembrolizumab concomitantly with WBRT and then continued with immunotherapy, who quickly responded to this therapeutic strategy and survived for 6 months following the initiation of systemic treatment.

## Acknowledgments

This study was supported by the OncoReSearch, Rovinka, Slovakia. The authors gratitude also go to Michael K. Hill, who helped improve the language and style of the final version of this manuscript.

## Author contributions

**Conceptualization:** Patrik Palacka.

**Data curation:** Palacka Patrik.

**Funding acquisition:** Patrik Palacka.

**Supervision:** Michal Mego.

**Writing – original draft:** Patrik Palacka, Jan Slopovsky, Marek Makovnik, Karol Kajo, Jana Obertova.

**Writing – review & editing:** Patrik Palacka, Michal Mego, Slopovsky Jan.
